# Telemedicine during the COVID‐19 epidemic improves outcomes in children with tuberous sclerosis complex: A 1206 visits retrospective cohort study

**DOI:** 10.1111/cns.14549

**Published:** 2023-11-30

**Authors:** Xia Wang, Ji Wang, Xiaonan Du, Lifei Yu, Yuanfeng Zhou, Shuizhen Zhou, Yi Wang, Yifeng Ding

**Affiliations:** ^1^ Department of Neonatology Xinhua Hospital Affiliated to Shanghai Jiaotong University School of Medicine Shanghai China; ^2^ Department of Neurology, National Children Medical Center Children's Hospital of Fudan University Shanghai China

**Keywords:** children, COVID‐19, epilepsy, telemedicine, tuberous sclerosis complex

## Abstract

**Aims:**

To evaluate the benefits of telemedicine in children with tuberous sclerosis complex during the COVID‐19 pandemic.

**Methods:**

A retrospective cohort study was conducted, comparing telemedicine and in‐person visits within the timeframe spanning from June 1, 2021, to June 1, 2022. Disparities in demographics, emergency visits, hospitalizations, adverse effects (AEs) associated with sirolimus, and the incidence of drug‐refractory epilepsy (DRE) between telehealth and in‐person care were assessed. Additionally, distinctions between audio and video telehealth, as well as varying frequencies of telehealth encounters, were investigated and reported as odds ratios (ORs).

**Results:**

A total of 378 patients with 1206 visits were included, of which 137 were telemedicine patients and 241 were in‐person patients. The median age was 5.0 years (IQR 2.8–10.0 years). There were 197 males (52.12%), 691 in‐person visits (57.30%), and 515 telemedicine visits (42.70%). Children under 12 years old, those farther away from the center, mothers with more than 12 years of education, and children treated with sirolimus were more likely to visit via telemedicine. Telehealth was associated with significantly fewer emergency visits, hospitalizations, AEs of sirolimus, and DRE. With 10 or more visits, the incidence of emergency visits, hospitalization, and DRE was significantly reduced.

**Conclusion:**

Telemedicine visits are almost as close in number as in‐person visits. Younger patients, patients in remote areas, and mothers with higher education levels are more willing to complete telemedicine visits. Telemedicine visits were associated with a significantly lower number of emergency visits, hospitalizations, and AEs of sirolimus. Patients with more than 10 visits per year seemed to have better clinical outcomes.

## Graphical abstract

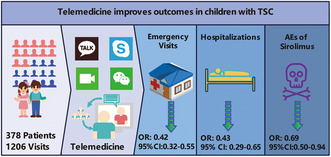

## 1 INTRODUCTION

The COVID‐19 outbreak, which began in late 2019 and led to widespread lockdowns in China and globally, has significantly increased the demand for telemedicine.^1^, ^2^, ^3^, ^4^ Patients with tuberous sclerosis complex (TSC), considered a prevalent rare disease, frequently need to visit neurology clinics for conditions like epilepsy, intracranial lesions, neuropsychiatric, and renal disorders.^5^, ^6^, ^7^, ^8^ China, a vast country with a significant population, has about 36.1% of its inhabitants residing in rural and remote regions. Local hospitals primarily offer general medical services, and patients with certain rare diseases often need to travel to major medical centers for specialized care. Children with TSC commonly experience epilepsy, intellectual disabilities, and renal lesions. Hence, regular clinical check‐ups and treatment adjustments are essential. Given this context, the Children's Hospital of Fudan University, National Children's Medical Center, a leading TSC treatment center in China, inaugurated its TSC Specialized Clinic in November 2017 to offer telemedicine services to children with TSC. The COVID‐19 outbreak highlighted the convenience and contactless advantages of these telemedicine services.

This study aimed to examine the determinants of telemedicine utilization at China's premier pediatric TSC center and assess its effects on clinical outcomes amidst the COVID‐19 pandemic.

## 2 METHODS

### 2.1 Subjects

**FIGURE 1:**
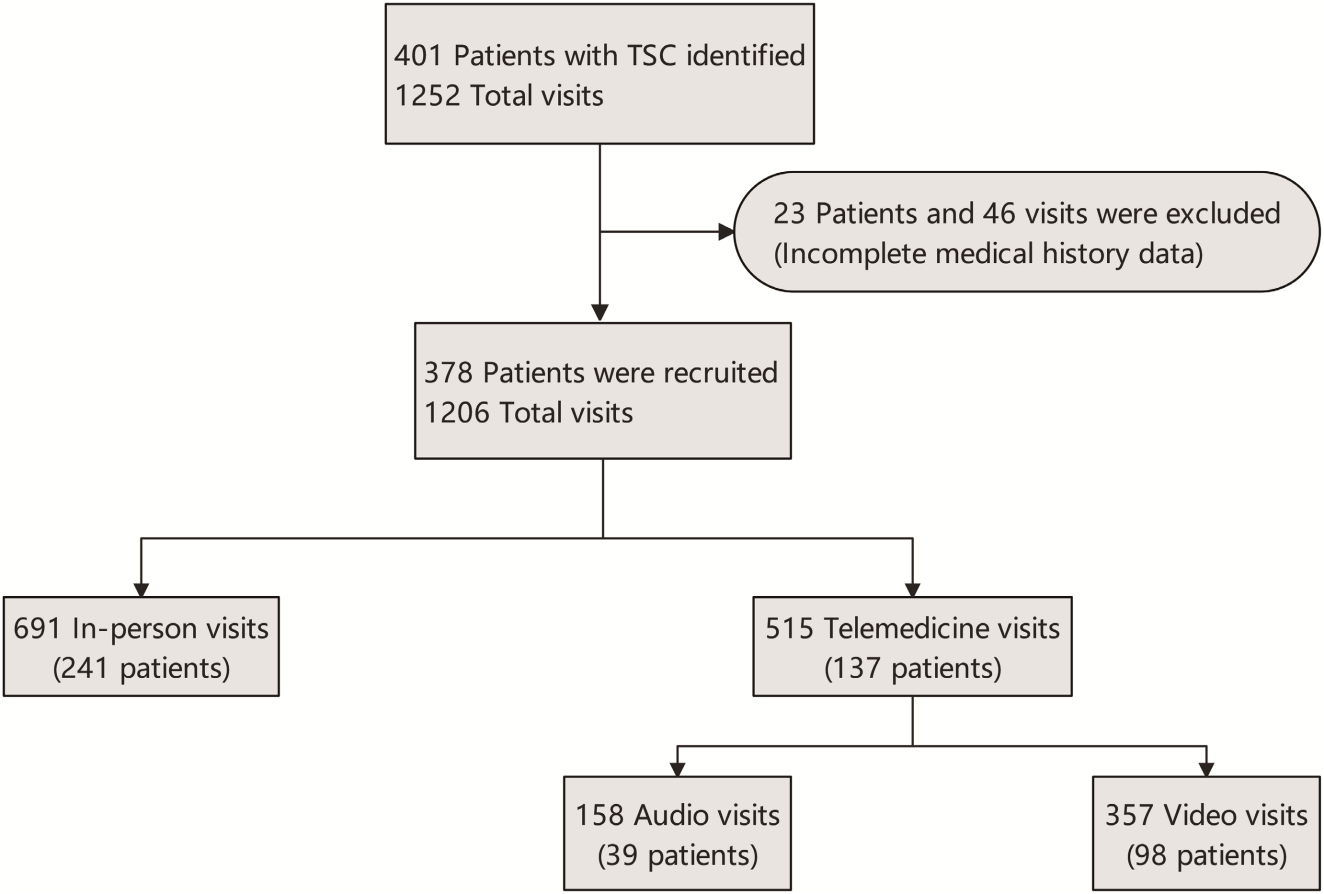
Flow diagram of the enrollment of children with tuberous sclerosis complex.

This was a retrospective study conducted by the Department of Neurology, Children's Hospital of Fudan University, National Children's Medical Center, which is one of the largest TSC diagnosis and treatment centers in China. The center was started in November 2013 and now provides 750–1500 visits per year. The center launched telemedicine services in November 2017 and provided QR codes for easy remote access. Patients can also access our center through various telemedicine software. Inclusion criteria included TSC‐diagnosed children who visited the pediatric neurology TSC specialty clinic of our hospital between June 1, 2021, and June 1, 2022 (Figure 1). Exclusion criteria included (1) non‐TSC children; (2) incomplete clinical data; (3) children who did not complete the entire remote or in‐person visit process. We define telemedicine visits as video visits or audio visits that the patient completes in their entirety using the video/audio function. Visits were excluded when the visit was conducted via video/audio but without a communication of the full condition (e.g., medication is dispensed, contacts do not know condition, incomplete medical information) or when the patient did not show up or missed the meeting. We do not conduct emergency telemedicine. Patient age, sex, genotype, habitual residence, concomitant epilepsy, use of sirolimus, adverse events (AEs) of sirolimus, emergency visit rate, and hospitalizations were investigated. The Ethics Committee of Children's Hospital of Fudan University, National Children's Medical Center approved the study and waived informed consent as the study was retrospective.

### 2.2 Implementation of telemedicine

Prior to the telemedicine consultation, the operator aids parents in uploading essential child data to the medical app. These data encompass name, gender, age, weight, hospital case number, test results for physician review, previous visit details, and primary objectives for the current consultation, potentially presented via text, imagery, or video. The appointment follows, enabling parent‐doctor communication through either audio, video, or text. All text and audio exchanges are available for unlimited review and replay.

### 2.3 Statistical analysis

Statistical analyses were conducted using JMP Pro v16.1.0 (SAS Institute Inc., USA). Variables not normally distributed were presented as medians with interquartile ranges (IQRs). The study assessed associations between telemedicine and various factors, including demographic elements, clinical data, genotype, epilepsy, and sirolimus usage. Additionally, the research evaluated associations of telemedicine with in‐person visits as well as the impact of audio/video consultations on clinical outcomes. The *χ* ^2^ or Fisher's exact test was employed for categorical variable analysis. Multivariate logistic regression analysis was utilized to determine telemedicine's correlation with clinical outcomes, accounting for the independence of patient visits and considering the previously mentioned demographic and clinical factors as potential covariates. A *p*‐value of <0.05 (two‐sided) was deemed statistically significant.

## 3 RESULTS

### 3.1 Demographic data

A total of 401 TSC patients and 1252 total visits were identified (Figure 1). A total of 23 patients and 46 visits were excluded due to incomplete medical history data. Of the remaining 378 patients, 137 were telemedicine patients and 241 were in‐person patients. Of the 1206 visits ultimately included, 691 (57.30%) were in‐person and 515 cases (42.70%) were telemedicine.

As shown in Table 1, the median age of the 378 patients included in the analysis was 5.0 years (IQR 2.8–10.0 years). The largest number of patients (201 [53.18%]) were in the childhood age range of 3–12 years. Of the patients, 197 were male (52.12%), 193 (51.06%) lived in suburban or rural areas, 190 (50.26%) came from within 300 km of the center, more parents of children with TSC had less than 9 years of education (fathers, 140 [37.04%]; mothers, 139 [36.77%]), and the highest monthly household income was in the range of RMB 5000–1000 (148 [39.15%]). Children with the *TSC2* gene variant accounted for the majority (249 [65.87%]), 308 (81.48%) of the children had comorbid epilepsy and 245 (64.81%) of the children were treated with sirolimus. Table 1 provides statistics on the relationship between demographic factors and the odds of telemedicine visits (expressed as ORs). Children under 12 years of age (<3 years, OR, 2.97, 95% CI, 2.00–4.39; 3–12 years, OR, 2.83, 95% CI, 1.97–4.07), those farther away from the center (300–700, 700–1500, ≥1500 km; OR, 4.71, 95% CI, 3.53–6.28; OR, 2.12, 95% CI, 1.49–3.01; OR, 5.79, 95% CI, 3.77–8.89, respectively), mothers with more than 12 years of education (OR, 1.39; 95% CI, 1.05–1.82) and those treated with sirolimus (OR, 2.21; 95% CI, 1.71–2.86) were more likely to visit via telemedicine, and we were surprised to find lower odds of telemedicine for patients with comorbid epilepsy (OR, 0.60; 95% CI, 0.44–0.81), while other demographic data were not significantly different.

**TABLE 1:** Demographic data and ORs of telemedicine vs. in‐person visits.

ategory	Unique patients no. (%) (*N* = 378)	Visits, no./total no. (*N* = 1206)		OR for telemedicine (95% CI)
		Telemedicine (*n* = 515)	In‐person (*n* = 691)	
Age, year				
<3 years	102 (26.98)	158/334 (47.31)	176/334 (52.69)	2.97 (2.00–4.39)
3–12 years	201 (53.18)	311/674 (46.14)	363/674 (53.86)	2.83 (1.97–4.07)
>12 years	75 (19.84)	46/198 (23.23)	152/198 (76.77)	1 [Reference]
Sex				
Female	181 (47.88)	239/587 (40.72)	348/587 (59.28)	1 [Reference]
Male	197 (52.12)	276/619 (44.59)	343/619 (55.41)	1.17 (0.93–1.47)
Place of residence				
Suburban or rural	193 (51.06)	252/609 (41.38)	357/609 (58.62)	0.90 (0.71–1.13)
Urban	185 (48.94)	263/597 (44.05)	334/597 (55.95)	1 [Reference]
Radial distance, km				
<300	190 (50.26)	143/557 (25.67)	414/557 (74.33)	1 [Reference]
300–700	90 (23.81)	218/352 (61.93)	134/352 (38.07)	4.71 (3.53–6.28)
700–1500	61 (16.14)	76/180 (42.22)	104/180 (57.78)	2.12 (1.49–3.01)
≥1500	37 (9.79)	78/117 (66.67)	39/117 (33.33)	5.79 (3.77–8.89)
Paternal education, year				
≤9	140 (37.04)	164/410 (40.00)	246/410 (60.00)	1 [Reference]
9–12	115 (30.42)	180/391 (46.04)	211/391 (53.96)	1.27 (0.97–1.69)
>12	123 (32.54)	171/405 (42.22)	234/405 (57.78)	1.10 (0.83–1.45)
Maternal education, year				
≤9	139 (36.77)	167/437 (38.22)	270/437 (61.78)	1 [Reference]
9–12	114 (30.16)	162/366 (44.26)	204/366 (55.74)	1.28 (0.97–1.70)
>12	125 (33.07)	186/403 (46.15)	217/403 (53.85)	1.39 (1.05–1.82)
Family monthly income, RMB				
<5000	115 (30.42)	143/349 (40.97)	206/349 (59.03)	1 [Reference]
5000–10,000	148 (39.15)	213/482 (44.19)	269/482 (55.81)	1.14 (0.86–1.51)
>10,000	115 (30.42)	159/375 (42.40)	216/375 (57.60)	1.06 (0.79–1.43)
Pathogenic variants in gene				
*TSC1*	86 (22.75)	135/310 (43.55)	175/310 (56.45)	0.81 (0.50–1.32)
*TSC2*	249 (65.87)	340/814 (41.77)	474/814 (58.23)	0.75 (0.48–1.19)
NMI	43 (11.38)	40/82 (48.78)	42/82 (51.22)	1 [Reference]
Epilepsy				
Yes	308 (81.48)	406/1001 (40.56)	595/1001 (59.44)	0.60 (0.44–0.81)
No	70 (18.52)	109/205 (53.17)	96/205 (46.83)	1 [Reference]
Sirolimus				
Yes	245 (64.81)	397/814 (48.77)	417/814 (51.23)	2.21 (1.71–2.86)
No	133 (35.19)	118/392 (30.10)	274/392 (69.90)	1 [Reference]

Abbreviations: CI, confidence interval; OR, odds ratio.

### 3.2 Association of visit patterns with clinical outcomes

As shown in Table 2, the emergency visit rates, AEs of sirolimus, hospitalization rates, and drug‐refractory epilepsy (DRE) rates were stratified by in‐person visits and telemedicine visits.

**TABLE 2:** Association of telemedicine visits with clinical outcomes.

Visit patterns	Total visits	Emergency visits		Hospitalization events		AE of sirolimus		DRE	
	No. (%)	No. (%)	OR^a^ (95% CI)	No. (%)	OR^a^ (95% CI)	No. (%)	OR^a^ (95% CI)	No. (%)	OR^a^ (95% CI)
Telemedicine vs. in‐person visits									
In‐person visits	691 (57.30)	298 (43.13)	1 [Reference]	116 (16.79)	1[Reference]	238 (57.07)	1 [Reference]	335 (56.30)	1 [Reference]
Telemedicine visits	515 (42.70)	120 (23.30)	0.42 (0.32–0.55)	41 (7.96)	0.43 (0.29–0.65)	161 (40.55)	0.69 (0.50–0.94)	200 (49.26)	0.74 (0.56–0.97)
Telemedicine visits									
Audio visits	158 (30.68)	50 (31.65)	1[Reference]	15 (9.49)	1[Reference]	44 (34.11)	1[Reference]	53 (46.49)	1[Reference]
Video visits	357 (69.32)	70 (19.61)	0.47 (0.30–0.76)	26 (7.28)	1.58 (0.70–3.55)	117 (43.66)	1.96 (1.17–3.27)	147 (50.34)	0.58 (0.34–1.00)
Frequency of telemedicine visits									
<5	215 (41.75)	61 (28.37)	1 [Reference]	25 (11.63)	1 [Reference]	59 (38.56)	1 [Reference]	108 (64.67)	1 [Reference]
5–10	109 (21.17)	37 (33.94)	1.36 (0.79–2.36)	5 (4.59)	0.24 (0.07–0.76)	31 (40.26)	1.08 (0.56–2.08)	38 (43.68)	0.33 (0.18–0.61)
≥10	191 (37.09)	22 (11.52)	0.20 (0.10–0.39)	11 (5.76)	0.22 (0.07–0.67)	71 (42.51)	1.38 (0.79–2.39)	54 (35.53)	0.28 (0.15–0.53)

Abbreviations: AE, adverse effect; CI, confidence interval; DRE, drug‐refractory epilepsy; OR, odds ratio.

^a^ Adjusted by the demographic and clinical variables as potential adjustment variables.

Compared with in‐person visits, telemedicine visits were significantly lower in emergency visits (OR, 0.42; 95% CI, 0.32–0.55), hospitalization (OR, 0.43; 95% CI, 0.29–0.65), AEs of sirolimus (OR, 0.69; 95% CI, 0.50–0.94), and DRE (OR, 0.74; 95% CI, 0.56–0.97).

Further comparison of audio and video visits revealed no significant differences in the rate of hospitalization (OR, 1.58; 95% CI, 0.70–3.55) or DRE (OR, 0.58; 95% CI, 0.34–1.00), but video appeared to be associated with emergency visits (OR, 0.47; 95% CI, 0.30–0.76) and more AEs of sirolimus events (OR, 1.96; 95% CI, 1.17–3.27).

Among telemedicine visits, a stratified analysis of the number of consultations found that with 10 or more visits, the incidence of emergency visits (OR, 0.20; 95% CI, 0.10–0.39), hospitalization (OR, 0.22; 95% CI, 0.07–0.67), and drug‐refractory epilepsy (OR, 0.28; 95% CI, 0.15–0.53) would be significantly reduced, even with 5 or more hospitalizations (OR, 0.24; 95% CI, 0.07–0.76) and DRE (OR, 0.33; 95% CI, 0.0.18–0.61), but an increase in the number of consultations did not seem to reduce the incidence of AEs of sirolimus (5–10 visits, OR, 1.08; 95% CI, 0.56–2.08; ≥10 visits, OR, 1.38; 95% CI, 0.79–2.39).

## 4 DISCUSSION

To our knowledge, this is the first study on the application of telemedicine in children with TSC during the COVID‐19 pandemic. We retrospectively summarize the current status of telemedicine implementation in China's largest children's TSC center. Factors that may influence telemedicine completion were analyzed, and the different effects on clinical outcomes between those participating or not in telemedicine (in‐person visits) were compared. This will provide a good trial and preliminary summary of nonface‐to‐face medical care under extreme public events and provide a basis for the formulation of public policies.

The COVID‐19 outbreaks in Wuhan, China, in late 2019 and in Shanghai in March 2022 have had a profound and widespread impact on individuals' daily lives.^3^, ^9^ Stringent travel restrictions were imposed on patients, and a significant portion of the population harbored concerns about potential infection when seeking healthcare, leading to a decline in patient numbers.^10^, ^11^ It was during this period that telemedicine consultations saw a notable surge in demand. Between March and June 2022, our TSC center, which had operated in a conventional in‐person model, was compelled to suspend its operations. Consequently, telemedicine services, initiated in 2017, assumed the primary role in patient follow‐up. We took proactive measures to promote telemedicine as a viable alternative, offering comprehensive training programs. The response from the vast majority of our patients was highly encouraging. Telemedicine, on the one hand, mitigates the risk of COVID‐19 transmission by enabling social distancing and reducing mobility. Simultaneously, it expedites care delivery, facilitates standardized monitoring of TSC‐associated neurological and psychiatric chronic conditions, and streamlines post‐hospitalization follow‐up. It also enhances access to TSC specialists, especially for patients facing mobility constraints or those residing at considerable distances, all while reducing the financial burden on patients.^12^, ^13^, ^14^

Our research reveals that, in the past year, the volume of telemedicine visits closely approximates that of in‐person visits. Notably, younger patients, individuals residing in remote geographical locations, and mothers with higher levels of education exhibit a greater inclination toward completing telemedicine consultations. Several factors may underlie these trends.

First and foremost, epilepsy in patients with tuberous sclerosis complex (TSC) typically manifests before the age of 2, necessitating more frequent visits for treatment plan adjustments and the monitoring of drug efficacy and side effects. Moreover, during the pandemic, the implementation of isolation policies in various regions rendered transportation a formidable barrier to healthcare access for children with TSC. Consequently, the demand for telemedicine increases proportionally with the distance from the medical center. Additionally, telemedicine often mandates the use of advanced electronic equipment and robust network connectivity, while the utilization of certain telemedicine applications necessitates a degree of technological literacy. Hence, a higher level of education facilitates the successful completion of telemedicine consultations.

However, some studies have suggested that telephone‐only visits may miss important clinical information and therefore be classified as unsuccessful telemedicine. However, our audio visits are guided by staff beforehand for patients to upload relevant video, audio, and text material, and the audio communication with the doctor takes place afterward, so we, like most other studies, believe that audio visits are also a very important method in telemedicine, which does not have strict requirements on network bandwidth and is sometimes more easily accepted by patients. In our study, we also found that audio and video visits were almost equally important, and both had better clinical outcomes (fewer emergency visits, less hospitalization, and fewer AEs of sirolimus) than in‐person visits without telemedicine. The benefits of video visits appear to be more pronounced in terms of emergency visits and may be recommended as a priority in telemedicine training.

This study assesses the clinical benefits associated with varying frequencies of telemedicine visits, an area with limited prior research. Patients receive tailored follow‐up frequencies based on their condition, and they have the option to request increased follow‐up; however, some may opt for reduced follow‐up. Excessive visits would impose a financial burden on patients and strain hospital telemedicine resources, whereas infrequent visits might not adequately address clinical care requirements. Our study identified improved clinical outcomes in TSC patients who had more than 10 annual visits. Consequently, we recommend patients to judiciously augment their telemedicine visit frequency.

During the COVID‐19 pandemic, telemedicine has emerged as an essential tool, with studies indicating that tele‐neurology is as effective as in‐person consultations.^15^ However, telemedicine, being in its nascent stage, has its imperfections. One primary limitation of telemedicine is its challenge in obtaining detailed phenotypic data via professional physical examinations. A prior study highlighted conditions that necessitate in‐person consultations, particularly for comprehensive eye exams, vestibular system assessments, muscle tone and reflex evaluations, and sensory testing.^16^ There is a considerable risk of delayed or missed diagnoses. Furthermore, while telemedicine offers many advantages, the nuances of in‐person interactions, such as conveying empathy and understanding, remain crucial in the doctor‐patient relationship. Immediate in‐person evaluations are essential for acute symptom exacerbations, including sudden seizure escalations, respiratory issues from lymphangioleiomyomatosis, or potential renal angiomyolipoma bleeding. For patients under consideration for procedures like epilepsy surgery or angiomyolipoma removal, direct consultations with the surgical team are vital. Conversely, the current self‐pay model for TSC‐specific telemedicine restricts access for some patients. Nonetheless, the hospital is in the process of integrating its platform with the health insurance system. With telemedicine, patients can undergo procedures like cranial MRI and video EEG at their local hospitals. Patient privacy protection remains paramount. We've addressed the concern of allowing patients to revisit the doctor's recommendations while ensuring exclusive access for the patient and the attending physician. Telemedicine offers consistent benefits, irrespective of public health crises. The COVID‐19 pandemic has heightened our recognition of its advantages. Initially a voluntary approach with limited traceability, telemedicine has evolved into a structured system, promising continued benefits for pediatric patient's post‐pandemic.^17^

During the COVID‐19 outbreak, the demand for telemedicine services surged dramatically due to various lockdowns and travel restrictions. Many patients avoided visiting hospitals for fear of contracting COVID‐19, making telemedicine their preferred choice. Additionally, with medical resources primarily focused on combating the pandemic, regular face‐to‐face medical services might have been limited, further propelling the use of telemedicine. In non‐epidemic periods, while telemedicine services still exist and are utilized by many patients, they primarily serve as a supplement to traditional medical services. Patients might opt for telemedicine services for convenience, especially those residing in remote areas or those who find it challenging to visit hospitals. However, in non‐pandemic times, telemedicine is not chosen out of urgency but more as an option or supplement.

Our study has several limitations. Although we promoted access to telemedicine by all possible means, telemedicine visits for children with TSC were not widely publicized by the government, and this does not exclude patients who had a need for teleconsultation but were not aware of the televised methods. Furthermore, this is a single‐center study, and although our center is one of the largest clinical and research centers for TSC in China and the population is representative, future multicenter studies are needed to further improve the representativeness of the sample. We believe that with the further improvement of telemedicine in our center and the support of the government, the results of our future long‐term study will be more objectively evaluated.

## 5 CONCLUSION

In conclusion, this study is the first to evaluate the application of telemedicine in pediatric neurology in China, demonstrating the benefits of telemedicine during public emergencies. This provides basic data for the formulation of public medical policies under the normalized prevention and control measures of China's COVID‐19 epidemic. In the context of global digitalization, telemedicine will surely become a necessary supplement to in‐person medical care. This has the potential to lead to a reallocation of medical resources among regions.

## FUNDING INFORMATION

This research was funded by the National Natural Science Foundation of China (no. 81803256).

## CONFLICT OF INTEREST STATEMENT

The authors declare no conflict of interest.

## Data Availability

The datasets used and/or analyzed during the current study are available upon request to the corresponding author.
